# Applications of Synthetic Biotechnology on Carbon Neutrality Research: A Review on Electrically Driven Microbial and Enzyme Engineering

**DOI:** 10.3389/fbioe.2022.826008

**Published:** 2022-01-25

**Authors:** Xiaoyan Zhuang, Yonghui Zhang, An-Feng Xiao, Aihui Zhang, Baishan Fang

**Affiliations:** ^1^ College of Food and Biology Engineering, Jimei University, Xiamen, China; ^2^ Department of Chemical and Biochemical Engineering, College of Chemistry and Chemical Engineering, Xiamen University, Xiamen, China

**Keywords:** carbon neutrality, enzyme engineering, electrically driven microbial, carbon metabolic pathway, synthetic biotechnology

## Abstract

With the advancement of science, technology, and productivity, the rapid development of industrial production, transportation, and the exploitation of fossil fuels has gradually led to the accumulation of greenhouse gases and deterioration of global warming. Carbon neutrality is a balance between absorption and emissions achieved by minimizing carbon dioxide (CO_2_) emissions from human social productive activity through a series of initiatives, including energy substitution and energy efficiency improvement. Then CO_2_ was offset through forest carbon sequestration and captured at last. Therefore, efficiently reducing CO_2_ emissions and enhancing CO_2_ capture are a matter of great urgency. Because many species have the natural CO_2_ capture properties, more and more scientists focus their attention on developing the biological carbon sequestration technique and further combine with synthetic biotechnology and electricity. In this article, the advances of the synthetic biotechnology method for the most promising organisms were reviewed, such as cyanobacteria, *Escherichia coli*, and yeast, in which the metabolic pathways were reconstructed to enhance the efficiency of CO_2_ capture and product synthesis. Furthermore, the electrically driven microbial and enzyme engineering processes are also summarized, in which the critical role and principle of electricity in the process of CO_2_ capture are canvassed. This review provides detailed summary and analysis of CO_2_ capture through synthetic biotechnology, which also pave the way for implementing electrically driven combined strategies.

## Introduction

With the advancement of science, technology, and productivity, the rapid development of industrial production, transportation, and the exploitation of fossil fuels has gradually led to the accumulation of greenhouse gases and deterioration of global warming (Fang et al., 2021). Global CO_2_ emissions have increased by 30.7% after humankind entered the 21st century. Supported by investigation report, if the carbon is still growing at such a high rate, the global concentration of CO_2_ will reach up to 5*10^^−4^ μl/L by 2050, leading to the extinction of 24% of animals and plants on the Earth (Pacala and Socolow, 2018; Johnson et al., 2021). It also has attracted the attention of countries worldwide that put goals of energy conservation, emission reduction, and carbon neutrality on the agenda (Zou et al., 2021). Carbon neutrality is a balance between absorptions and emissions achieved by minimizing CO_2_ emissions from human social productive activity through a series of initiatives, including energy substitution and energy efficiency improvement. Then CO_2_ was offset through forest carbon sequestration and captured at last (He et al., 2021). At present, the routes of chemistry (Kourosh et al., 2020), electrochemistry (Li FH et al., 2020), photoelectric catalysis (Wang and Song, 2020), enzyme (Yang et al., 2020), and microbial carbon fixation (Hu et al., 2019) are widely studied. Compared with the chemical route, the biological route, which does not require high temperature and pressure, is a more environmentally friendly process.

**FIGURE 1:**
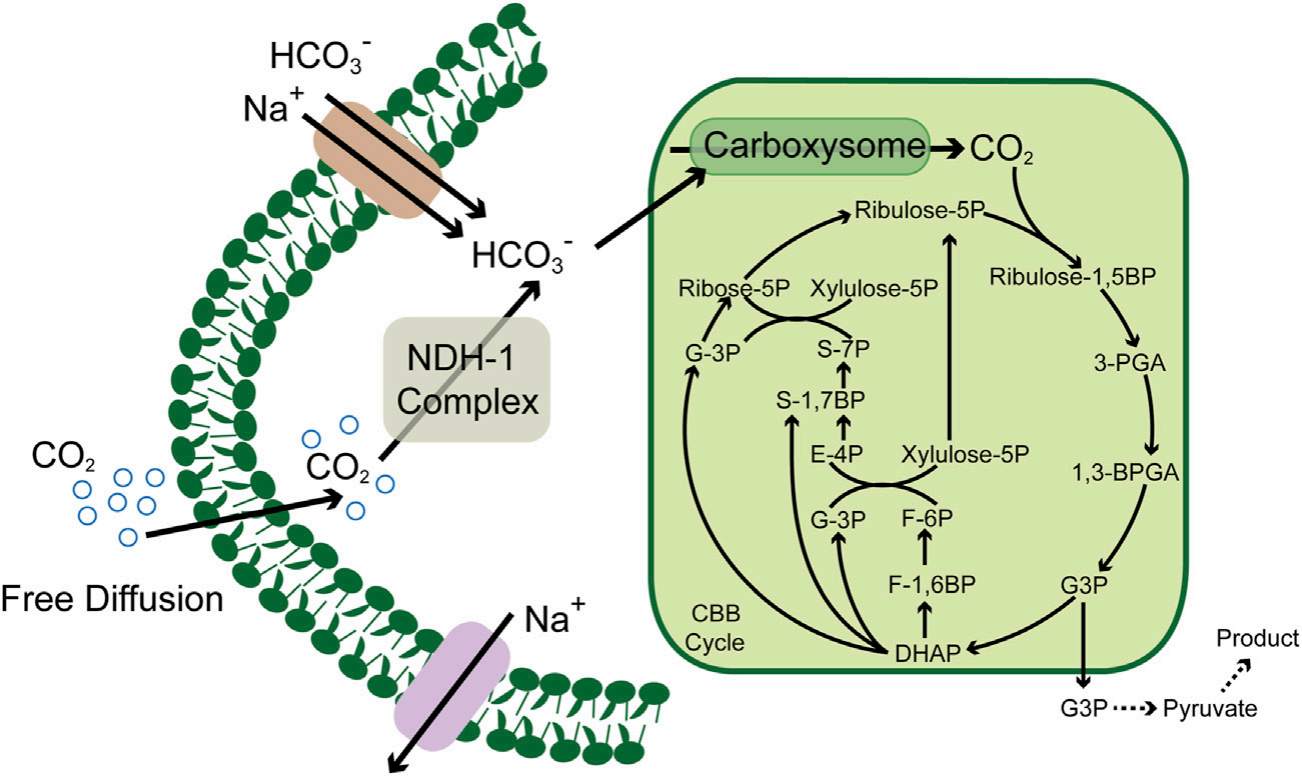
Schematic of the carbon concentrating mechanism in cyanobacteria. CBB cycle, Calvin–Benson–Bassham cycle; ribulose-1, 5BP, ribulose-1, 5 diphosphate; ribulose-5P, ribulose-5 phosphate; ribose-5P, ribose-5 phosphate; 3PGA, glycerate 3-phosphate; 1, 3BPGA, 1,3-bisphosphoglycerate; G3P, glyceraldehyde 3 phosphate; DHAP, dihydroxyacetone phosphate; F-1, 6BP, fructose-1, 6 diphosphate; F-6P, fructose-6 phosphate; xylulose-5P, xylulose-5 phosphate; S-7P, sedoheptulose-7 phosphate; S-1,7BP, sedoheptulose-7 diphosphate; and E-4P, erythrose-4 phosphate.

CO_2_ has played a vital role in the origin of life (Zhang et al., 2020), in which many species could fix as a carbon resource and flow into the metabolic pathways (Cheah et al., 2016). The central CO_2_-involved pathways are the Calvin cycle, reducing citric acid cycle, Wood–Ljungdahl pathway, 3-hydroxypropionic acid cycle, 3-hydroxypropionic acid/4-hydroxybutyric acid cycle, and the dicarboxylic acid/4-hydroxybutyric acid cycle (Aresta et al., 2016). These cycles have maintained the global carbon balance between absorptions and emissions in the past billions of years. However, with the economic development and environmental changes, the CO_2_ emission rate has far outstripped the traditional carbon fixation, and the balance was broken, which also declares the urgency to explore effective means to reduce CO_2_. Enzymes play an essential role in the microbial carbon fixation process, which also exert considerable influence on the CO_2_ fixation pathways; for example, the irreplaceable role of ribulose-1,5-bisphosphate carboxylase/oxygenase (RuBisCO) (Bhat et al., 2017) and formate dehydrogenase (Kumar et al., 2017) in the Calvin cycle (Figure 1) and the Wood–Ljungdahl pathway, respectively. Thus, the key enzymes and metabolic pathways involved in the existing CO_2_ metabolic pathways provide essential references for developing and enhancing enzymatic conversion and microbial fixation of CO_2_ (Jiang et al., 2021; Nisar et al., 2021; Tan and Ng, 2021).

In recent years, initial success has been achieved in the key enzymes’ structural design and modification, cofactor engineering, and metabolic engineering to improve efficiency (Chida et al., 2007; Fast and Papoutsakis, 2018). Moreover, advances and innovations of synthetic biotechnology and electrically driven microbial processes have given a new impetus for carbon neutrality.

In this study, we have introduced the natural and artificially modified microbial CO_2_ fixation progresses through synthetic biotechnology, in which CO_2_ was used as the carbon source for growth and product synthesis. And then, we also discussed the electrically driven enzymatic and non-autotrophic microbial CO_2_ fixation process, including mechanism, method, and important progress. Finally, based on full insights into the advantages and disadvantages of synthetic biotechnology and electrochemistry, we prospected the tendency development in the scope of the combination of synthetic biotechnology and electrochemistry, which is expected to provide a new solution for the efficient utilization of CO_2_ to generate high value–added chemicals.

## Synthetic Biotechnology for Carbon Neutrality

### Carbon Dioxide Fixation Through Cyanobacteria

Cyanobacteria are a kind of immemorial autotrophic prokaryotic organism with records from over 1 billion year ago, which makes a system to convert the CO_2_ to glucose, produce O_2_ from H_2_O, and create a favorable atmospheric environment for the formation of the wide range of living organisms. Cyanobacteria can synthesize various kinds of natural compounds from CO_2_ and energy absorbed from sunlight, for example, amino acids, pigment, and fatty acids (Lau et al., 2015). Compared with plants, the fast-growing and efficient CO_2_ conversion bacteria, cyanobacteria, have taken the duty of CO_2_ capture on its shoulder since ancient times. They also hold promise of being a gifted chassis for a cell factory of chemicals modified through synthetic biotechnology toolboxes. However, compared with model organisms of *E. coli* and yeast, the limitation of genetic manipulation of cyanobacteria cannot be ignored. Various significant efforts have developed an utterly robust set of synthetic biotechnology toolboxes and modularly recombined parts. These tools include available promoters (Behle et al., 2020; Sengupta et al., 2020), ribosome-binding sites, vector systems, and the CRISPR-Cas system, which are also reviewed comprehensively by Sun et al. (2018); Santos-Merino et al. (2019); Pattharaprachayakul et al. (2020); Santos-Merino et al. (2021). Beyond that, recombinant protein stability was also another crucial factor that decided the outcome of the heterologous expression. The recombinant proteins derived from eukaryotic plants and animals are unstable when freely expressed in the cyanobacterial cytosol but stable when fused with a highly expressed cyanobacterial native or heterologous protein, which was demonstrated by expressing the recombinant proteins of the plant origin isoprenoid biosynthetic pathway, human interferon protein, and tetanus toxin fragment C (Zhang X et al., 2021). These fundamental research studies pave the way to construct a robust engineered cyanobacterial cell factory for the large-scale commercial production from CO_2_.

Based on these pioneering works, many researchers focus on shaping a versatile producer from cyanobacteria. Compared to various heterotrophic model organisms, the relatively lower growth and metabolic rate were the barriers that hindered the development of cyanobacteria from the source, which derived from a lower CO_2_ fixation rate. However, compared to many other autotrophic organisms, cyanobacteria have a highly efficient CO_2_ enrichment system, which enables them to survive the water environment with low concentrations of CO_2_. In this system, the NDH-1/NDH-1MSs complex converts the CO_2_ diffused freely into a cell to HCO_3_^-^, which is further converted to CO_2_ by carbonic anhydrase in carboxysome and provided to RuBisCO as substrate (Angermayr et al., 2015, Figure 1). This unique mechanism guaranteed the CO_2_ concentration gradient between the intracellular and extracellular environments. Thus, recent efforts have focused on expanding the wavelength range of the absorbable solar spectrum and increasing electron transport chain activity to increase photosynthetic efficiency. Researchers introduced the chlorophyll f (Chlf)–encoding genes into *Synechococcus* sp. PCC 7002 absorbs far-red light of wavelengths over 700 nm (Ho et al., 2016). After integrating Chlf into PSI complexes, the active radiation for the new PSI complex was expanded up to 750 nm, which extended the wavelength ranges and provided light compensation under non-saturating light conditions (Tros et al., 2020).

Meanwhile, overexpression enzymes related to the Calvin cycle can improve photosynthesis and product formation, which have been shown in several research reports (De Porcellinis et al., 2018; Liang et al., 2018). It was also demonstrated in *Synechocystis* PCC 6803, where extra bicarbonate transporter expression led to a 2-fold enhancement of the growth rate and a higher amount of biomass accumulation (Kamennaya et al., 2015). Likewise, the overexpression of the carbon transporters BicA and SbtA involved in central carbon metabolism enhances biomass production by 50–100% (Gupta et al., 2020). Beyond that, Włodarczyk and his colleague (Włodarczyk et al., 2020) have discovered and characterized a new cyanobacterial strain, *Synechococcus* sp. PCC 11901, which possesses a shorter doubling time (2 h) and higher biomass production. By engineering this strain, they demonstrated that this promising cyanobacterium was easy to modify and produced free fatty acids with a concentration over 6 mM (1.5 g/L). Ungerer et al. (Ungerer et al., 2018) further identified three specific genes, *atpA*, *ppnK*, and *rpaA*, with SNPs from the fastest growing cyanobacterium, in which *atpA* and *ppnK* express an ATP synthase and NAD^+^ kinase with higher performance, resulting in the decrease in the doubling time from 6.8 to 2.3 h. After point mutation in the α subunit of F_o_F_1_ ATP synthase (AtpA), they enhanced the environmental stress tolerance of *Synechococcus elongatus* PCC 7942, leading to an increase in AtpA protein levels, intracellular ATP synthase activity, and ATP concentrations (Lou et al., 2018).

Many scholars devote themselves to exploring cyanobacteria as a multifunctional platform for a biotechnological process by far. By the introduction of the exogenous glycerol biosynthetic pathway, researchers build a bridge from the CO_2_ fixation to glycerol production, which serves as the substrate for the C3 platform chemicals (Wang et al., 2015). Using it as a base, scientists synthesized a variety of chemicals from CO_2_, including 3-hydroxypropionic acid (Wang et al., 2016), isobutanol (Miao et al., 2017), limonene (Lin et al., 2017), and 2,3-butanediol (Nozzi et al., 2017). Beyond that, by introducing a more complicated pathway, costly compounds of polysaccharide terpene (Bhunia et al., 2018) and fatty acid ethyl esters were also produced from these engineered cell factories. By expressing the genes coding for sucrose-phosphate synthase, sucrose-phosphate phosphatase, and sucrose-degrading invertase, sucrose was synthesized and accumulated successfully (Kirsch et al., 2018; Vayenos et al., 2020.). By overexpressing ribDGEABHT (riboflavin-encoding genes) and introducing an internal promoter to the upstream of the heterologous ribAB gene, the production of riboflavin increased by 211-fold (73.9 ± 7.2 μM) compared to the wild-type strain (Kachel and Mack, 2020).

However, the growth and production processes based on photoautotrophic are limited to the time of sunlight available because growth and synthesis were slowed down and ceased in an unlighted environment. In addition, economic feasibility in the application of cyanobacterium as a versatile producer also relies partially on their photosynthetic capacity and solar energy conversion efficiency. The imbalances between absorbed light energy (source) and the metabolic capacity (sink) can potentially increase the carbon flux output from the Calvin cycle, which may be beneficial for increasing the photosynthesis efficiency (Santos-Merino et al., 2021). Many genetic manipulation tools and strategies (Sun et al., 2018) have been developed to translate to significant gains for cyanobacteria as a cell factory to synthesize commercial products from CO_2_. At the same time, it is still a heavy responsibility and a long way from the goal for carbon neutrality with higher efficiency.

### Carbon Dioxide Fixation Through *Escherichia coli*

**FIGURE 2:**
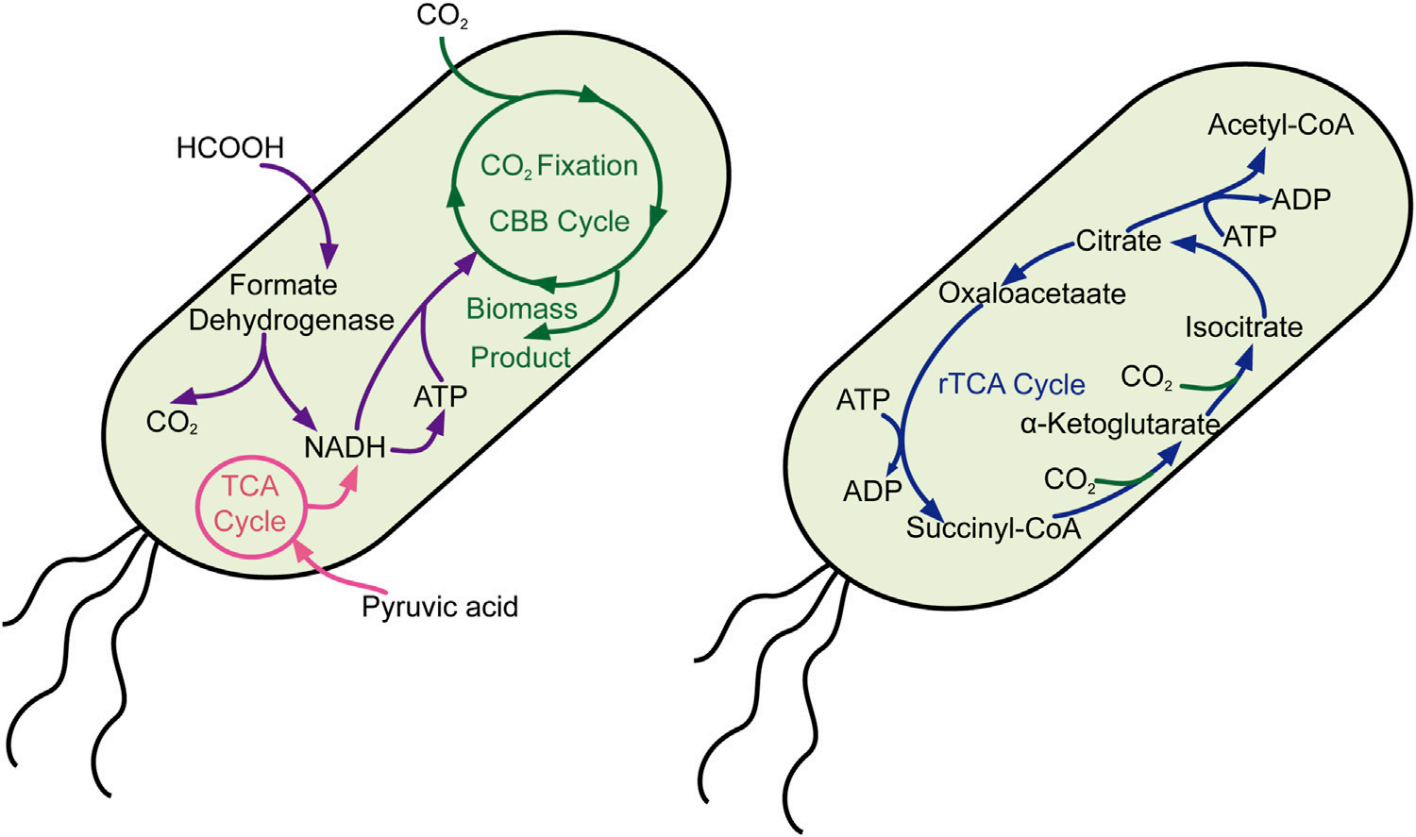
Carbon fixation in *E. coli* through the CBB and rTCA cycles. CBB cycle, Calvin–Benson–Bassham cycle; rTCA cycle, reverse citric acid cycle.

Unlike cyanobacteria, *Escherichia coli* is a normal chassis cell for most scientists who possess complete and efficient genetic manipulation tools. Thus, many scientists have devoted themselves to introducing the CO_2_-fixed pathway into *E. coli*, including the Calvin–Benson–Bassham cycle and the reductive tricarboxylic acid cycle. Milo’s group reported significant work that *E. coli* synthesized sugar from CO_2_ by introducing the non-native Calvin–Benson–Bassham cycle pathway (Figure 2), rewiring the metabolic pathway and directed laboratory evolution, while oxidization of pyruvate provided the reducing power and energy (Antonovsky et al., 2016). Moreover, they also constructed another engineered *E. coli* that use CO_2_ as their sole carbon source through metabolic rewiring and directed evolution, in which formate is oxidized to provide reducing power and energy (Gleizer et al., 2019). Lee and his colleague also heterogeneously expressed the whole gene clusters (cbbI and cbbII operons) belonging to the Calvin–Benson–Bassham (CBB) pathway in *E. coli*, which was combined with the yeast fermentation process to mitigate exogenous CO_2_. These milestone works provide feasible solutions in changing model heterotrophic organisms to autotrophy one and offer potential possibilities for resource sustainability.

Except for the CBB pathway, the scientist also reconstructed another C1 assimilation pathway to convert the nutritional types of *E. coli* for integrated utilization of CO_2_ and C1 chemical compounds. By employing the technique of rational design, metabolic pathway reconstruction, and metabolic flux rebalance, scientists achieve success in converting the *E. coli* to an engineered methylotrophic bacterium, which utilize methanol as a sole carbon source for growth with a doubling time of 8 h (Chen CH et al., 2020). By reconstructing the tetrahydrofolate cycle and the reverse glycine cleavage pathway and introducing formate dehydrogenase, the formate and CO_2_ could serve as carbon sources for engineered *E. coli*–sustaining growth (Bang and Lee, 2018). The combined reconstructed reductive glycine pathway and short-term evolution, Kim and his colleague also constructed an engineered *E. coli* by taking formate and CO_2_ as carbon sources, whose doubling time was less than 8 h. By introducing methanol dehydrogenase to the evolved strain, they also converted the engineered *E. coli* to a methylotrophic bacterium that could grow on methanol and CO_2_ (Kim et al., 2020).

Until now, it is hard to achieve the purpose of efficiency CO_2_ fixation from the liquid culture medium environment to produce chemical with high productivity, so reutilized CO_2_ from the endogenic pathway was particularly significant to reduce carbon emission in large-scale industrial production. By introducing the gene of *kor* (express α-ketoglutarate: ferredoxin oxidoreductase), *acl* (express ATP-dependent citrate lyase), *frd* (express fumarate reductase), and energy pump, the engineered *E. coli* successfully recycled CO_2_, in which the C-2/C-1 ratio increased to 1.79 ± 0.02 (Chen FYH et al., 2020). Beyond that, the scientists also introduced 20 genes related to the CO_2_-concentrating mechanism into *E. coli* to enhance the capture rate of CO_2_, which achieved success in fixing CO_2_ from ambient air into biomass (Flamholz et al., 2020). This work would let us not only understand the CO_2_-concentrating mechanism but also lay the groundwork for CO_2_ fixation in diverse organisms.

The research on *E. coli* that uses CO_2_ to produce high value–added products has achieved preliminary success. However, in contrast to cyanobacteria, *E. coli* is still growing slowly in autotrophic culture conditions. Therefore, to realize industrial-scale production, a large amount of work was still required to enhance CO_2_ fixation and utilization efficiency of engineered *E. coli*, which could also pave the way for the high value–added product.

### Carbon Dioxide Fixation Through Yeast

*Saccharomyces cerevisiae* is a versatile chassis cell which is widely used as a cell factory of natural compounds, especially for the industrial production of bioethanol. However, the anaerobic fermentation process constrains ethanol concentration and trigger the accumulation of by-product (glycerol), which is caused by the redox-cofactor unbalancing, Guadalupe-Medina select CO_2_ as an electron acceptor, which not only balances excess reduced cofactor (NADH) but also captures the CO_2_ and reduces greenhouse gas emissions (Guadalupe-Medina et al., 2013). They reconstruct a new pathway to introduce phosphoribulokinase (PRK) and form-II ribulose-1,5-bisphosphate carboxylase (Rubisco), leading to a result with lower (90% reduction) by-product and higher (10% increase) ethanol production. On this basis, Xia et al. studied the heterologous expression of the xylose reductase (XR)/xylitol dehydrogenase (XDH) and xylose isomerase, which converts xylose to xylulose (Xia et al., 2016). By introducing PRK and Rubisco and upregulating the native pentose phosphate pathway (PPP), they successfully achieved bioethanol production from cellulosic hydrolysates with CO_2_ recycling. Joeline Xiberras et al. introduce the “SA module” (malate dehydrogenase, fumarase, and fumarase) to *S. cerevisiae* for succinic acid production from glycerol and CO_2_ (Xiberras et al., 2020). These studies provide a feasible idea to fix CO_2_ and form other chemical compounds.

Unlike capturing CO_2_ to form chemical compounds through the reconstructed intracellular pathway, a biologically catalyzed CO_2_ mineralization process was another simple approach. Roberto Barbero et al. displayed bovine carbonic anhydrase II on the yeast’s surface, which has higher thermal stability and mineralized CO_2_ with coal fly ash to form CaCO_3_. Coupled with model prediction, they demonstrated that this biological mineralization process is ∼10% more cost-effective when captured per ton of CO_2_ (Barbero et al., 2013). Shen et al. treated the discarded yeast with potassium hydroxide and form microporous carbon materials with a Brunauer–Emmett–Teller surface area of 1,348 m^2^g^−1^ and a pore volume of 0.67 cm^3^g^-1^, which resulted in a superior performance for CO_2_ capture (Shen et al., 2012).

**FIGURE 3:**
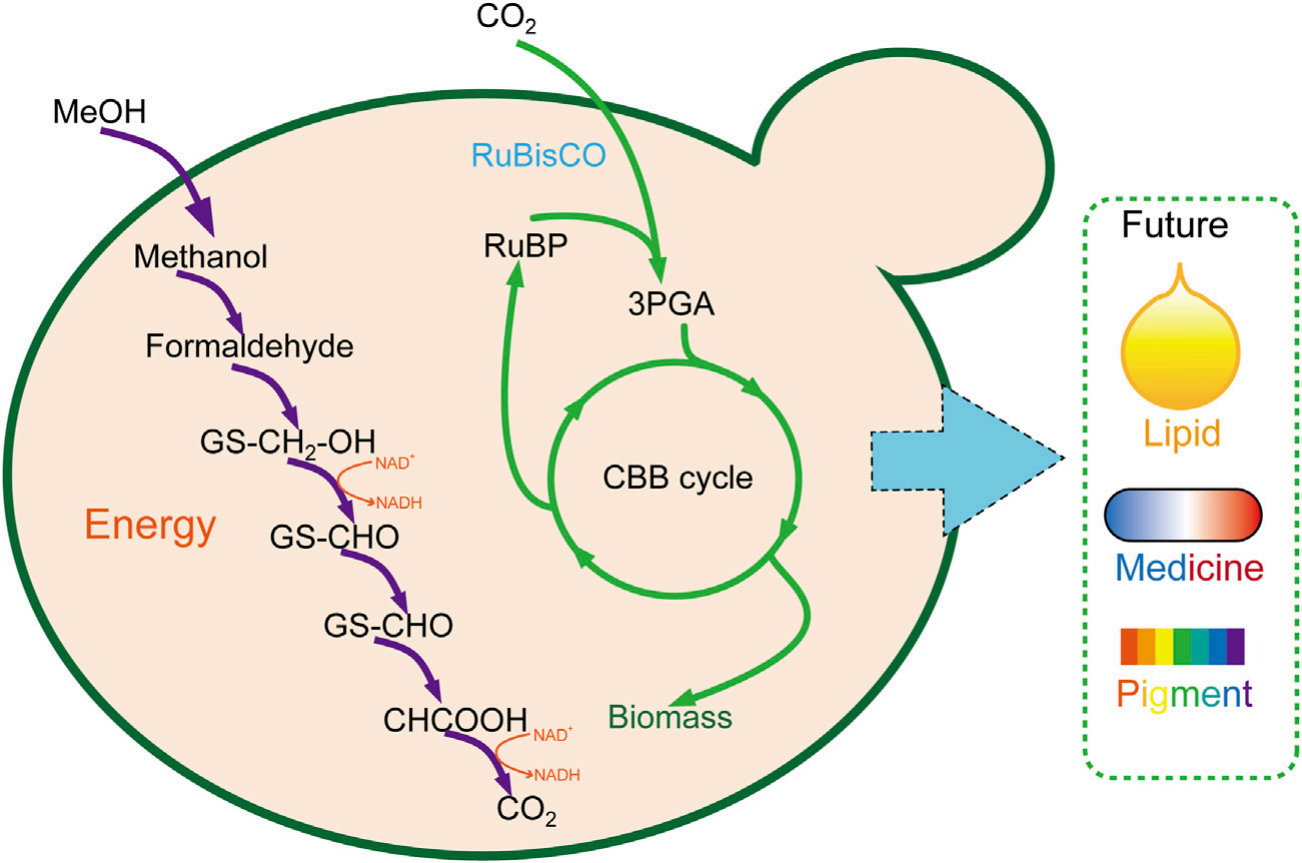
Carbon fixation in *P. pastoris* through the CBB cycle. 3PGA, glycerate 3-phosphate; CBB cycle, Calvin–Benson–Bassham cycle.

As eukaryotic chassis cells, yeast is more complicated and suitable for natural product synthesis than *E. coli*. Abundant genetic manipulation makes it more feasible to utilize CO_2_ to the product of high value–added long-chain compounds (Figure 3). However, as same as *E. coli*, a large amount of work was still required to enhance the CO_2_ fixation and utilization efficiency before being applied for large-scale industrial manufacture.

## Electrically Driven Carbon Neutrality

### Electron Transfer Mechanisms

**FIGURE 4:**
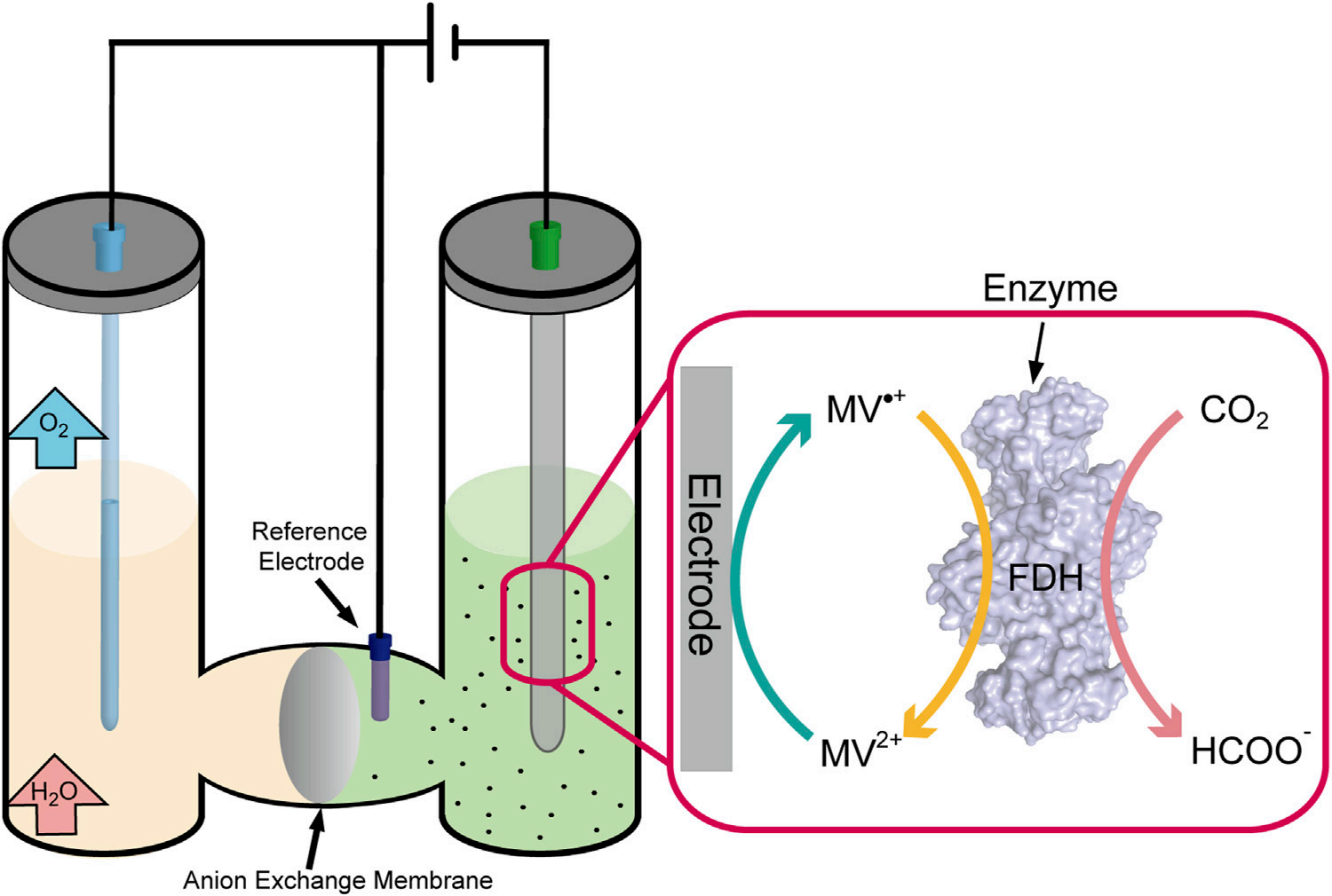
Schematic representation of the CO_2_ fixation process by electrically driven enzyme.

Enzyme catalytic CO_2_ fixation involves a redox reaction, in which electron transfers were performed by the cofactor of NAD(P)/NAD(P)H. However, the electrical process could realize the regeneration of electron acceptors and donors (cofactor) happened in electrodes to maintain the continuity of the reaction (Chen H et al., 2020). The electron across enzyme (e.g., cytochrome c and ferredoxin) can transfer directly from the electrode to the substrate when the enzyme’s active site is well exposed (Silveira and Almedia, 2013). Although many successes have been achieved in adsorption enzymes on the surface of the electrode, this process was unstable and not feasible in most situations that limit the movement process of the enzyme. However, the electron transfers mediately utilizing carriers (e.g., viologens, quinones, and dyes) shuttle out from the active site to the electrode surface, in which the active site resides deep inside the enzyme (Yuan et al., 2019). That seems more reasonable and practical in non-contact communications between enzymes and electrons, and the drawback is the toxicity from redox mediators (Figure 4).

**FIGURE 5:**
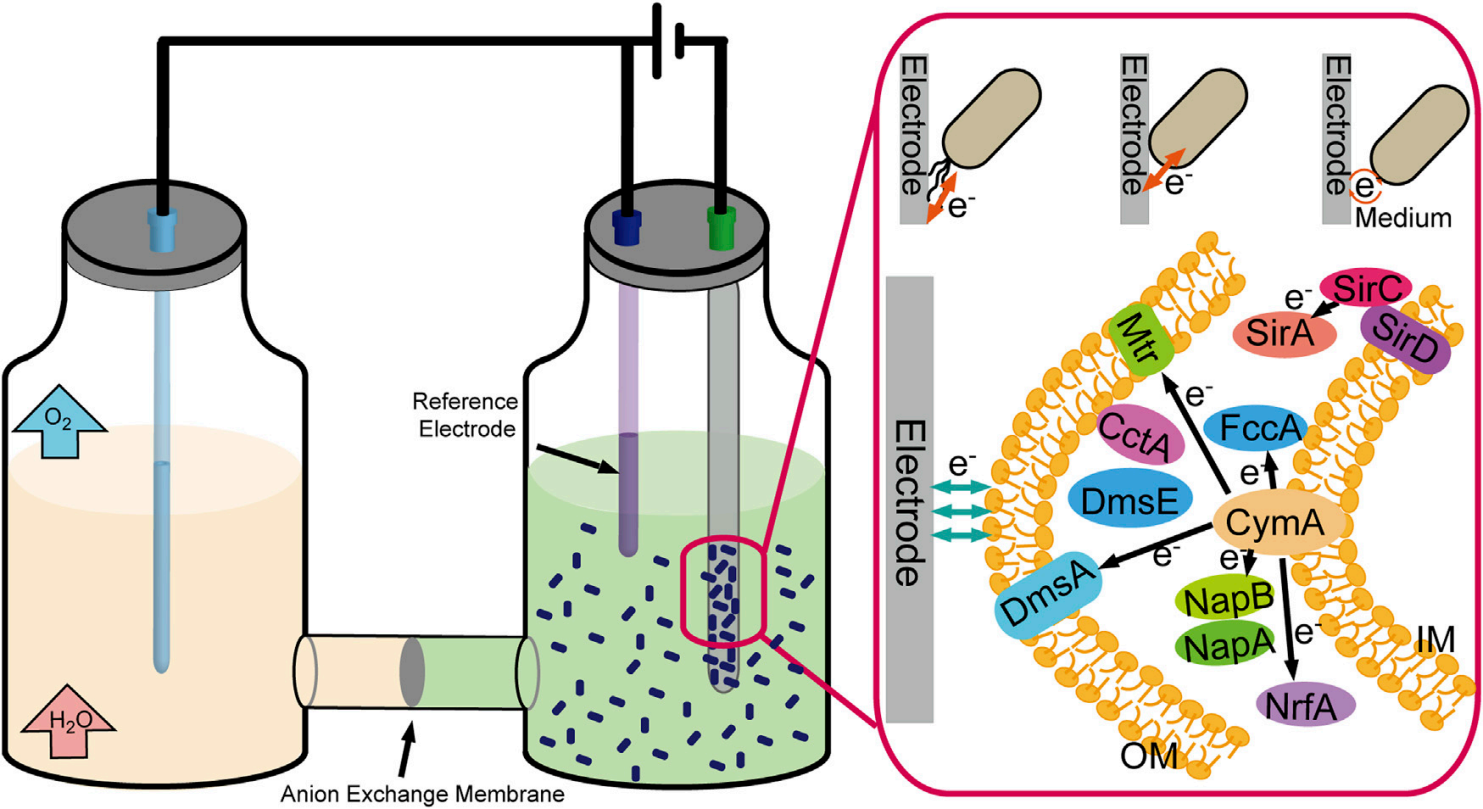
Schematic representation of the CO_2_ fixation process by electrically driven microorganism.

Some microbial cells can also replace enzymes to realize electron transfer with the electrode, such as *Shewanella oneidensis* and *Geobacter sulfurreducens*. There have been three significant mechanisms for electron transfer between the electrode and microbial cells (Figure 5). First, perform electron transfer by contacting the electrode immediately through c-type cytochromes located in the cell’s outer membrane; second, perform electron transfer through redox mediators to communicate with the electrode; and third, perform long-range electron transfer through pili (Pankratova et al., 2019). Compared with enzymes, the microbial cells possess the characteristics of high stability and self-duplicating and do not require purification belonging to the enzyme preparation process. Meanwhile, the weakness of lack of specificity and the slower electron transfer rate must not be neglected.

### CO_2_ Fixation by Electrically Driven Enzyme

#### CO_2_ Reduction to C1–C2 Chemical

CO_2_ fixation through enzymes was also lucubrated, which was generally performed through hydrogenation reduction and required an expensive cofactor of NADH/NADPH. Formate dehydrogenase (FDH) has been widely used in coenzyme regeneration, which catalyzes nearly irreversible formate oxidation to CO_2_ and provides NADH for the other coupling reaction. Metal-dependent FDH mainly catalyzed CO_2_ fixation to produce formate, using NAD^+^ as a cofactor. However, Jayathilake et al. developed a novel approach for CO_2_ reduction catalyzed by metal-independent FDH with methyl viologen radical cation (MV•+) as the cofactor, which efficiently regenerated at a carbon electrode through electrochemical reduction without any additional reducing agent (Figure 4). Formate yields as high as 97 ± 1% at 20 mV negative to the reversible electrode potential, much lower than that of metal catalysts (−800 mV to −1,000 mV) (Jayathilake et al., 2019). By embedding the enzymes into the metal–organic framework ZIF-8, Rh complex–grafted electrode was used to regenerate NADH and significantly enhanced the catalytic enzyme rate by 12-fold from CO_2_ to methanol compared to the free enzyme statue (Zhang Z et al., 2021). The enzyme catalytic CO_2_ fixation combined with electrochemical regeneration of natural/artificial cofactor provides a new idea for efficient CO_2_ fixation to small-molecule compounds.

#### CO_2_ Reduction to Higher Value–Added Products

Beyond that, the electrically driven enzyme also catalyzed CO_2_ to produce higher value–added products. Two cooperating enzymes in nanopores performed carboxylation by introducing CO_2_ to pyruvate (C3) and producing malate (C4), in which indispensable NADH was regenerated and driven by electricity (Morello et al., 2019). A bio-electrocatalytic system was also developed to drive carboxylation by incorporating CO_2_ into crotonyl-CoA, ferredoxin NADP^+^ reductase (FNR), and NADPH-dependent crotonyl-CoA carboxylase/reductase were co-immobilized in a viologen-based redox hydrogel. The faradaic efficiency was 92 ± 6% at a rate of 1.6 ± 0.4 μmol cm^−2^ h^−1^ (Castañeda-Losada et al., 2021). Thus, combined with the electrical method, the enzyme could be used for higher value–added products synthesis with high efficiency.

### CO_2_ Fixation by the Electrically Driven Microorganism

On the one hand, enzyme catalytic CO_2_ fixation result in high activity and selectivity, and on the other hand, the expensive protein purified process was also non-negligible. So, some scientists have devoted themselves to developing an electrically driven whole-cell catalytic process. *Methylobacterium extorquens* AM1 perform CO_2_ fixation to synthesize formate driven by the electrical method with product concentrations up to 60 mM. Compared to the electrically driven enzyme catalytic process, the whole-cell electro-biocatalytic process is undismayed by being exposed to oxygen gas without providing extra cofactors (Hwang et al., 2015). Employing neutral red as a redox mediator coated outside the carbon felt (CF) electrode, Seelajaroen et al. performed a long-term (17 weeks) electrical reduction of CO_2_ to formate based on *M. extorquens* (Seelajaroen et al., 2019). Beyond that, the bioelectrochemical system also harvests success in producing acetate, methane, butyrate, and polyhydroxybutyrate (PHB). Thus, CO_2_ fixation by electrically driven microorganisms is a process that converts renewable electrical energy to chemicals, which is a novel and attractive strategy for energy transformation and storage.

### CO_2_ Fixation Efficiency Between Electrically Driven CO_2_ Fixation and Bio-Carbon Fixation

It is hard to compare the CO_2_ fixation efficiency and rate between electrochemistry and bio-carbon fixation processes at “fair” levels because electric energy is converted from solar, wind, and chemical energy. However, we could also get some information and answers from interesting data. After the pioneer’s work, the heterotrophic microorganism (*E. coli*) was engineered to grow on methanol/formic acid and CO_2_, and the doubling time was decreased from ∼70 to ∼8 h. By contrast, the doubling time of *Synechococcus* sp. PCC 11901 (photosynthetic autotrophs), *E. coli* (chemoheterotrophy), and *Vibrio natriegens* (chemoheterotrophy) is 6–2 h (Włodarczyk et al., 2020), 27.23 min (Weinstock et al., 2016), and 15.61 min (Weinstock et al., 2016). Thus, there is a significant untapped opportunity for engineered microorganisms to synthesize products from CO_2_ with acceptable efficiency. In chemical synthesis, the electro-biocatalytic process produces 60 mM formate from CO_2_ in 60 h (1 mM/h) (Hwang et al., 2015) and 14.8 mM malate from pyruvate and CO_2_ in 24 h (0.62 mM/h) (Morello et al., 2019). Furthermore, CO_2_ was converted to starch at last with a rate ∼8.5-fold higher than starch synthesis in maize, in which electrically driven CO_2_ fixation was combined with the cell-free enzymatic catalytic process. Although electrically driven techniques have gained a temporary lead in efficiency, obstacles from large-scale industrialized production still stared them in the face.

## Combined Synthetic Biotechnology With Electrochemistry for Carbon Neutrality

By rewiring the metabolic pathway and introducing an exogenous gene module, the electroactive microbial cells can expand their product scope and electro-biocatalysis efficiency. However, there are a finite number of electroactivity microorganisms, and their gene modification platform was limited compared with the model organism. Moreover, the application of these electro-biocatalysts still faces much obstacles, for example, 1) the effect of the electrical environment on the formation of biofilms, 2) limited toolbox for genetic manipulation, and 3) low extracellular electron transfer rate. So, we take *S. oneidensis* as a representative, which has been studied maturely and in-depth relatively.

### Facilitate Biofilm Formation

Electroactive microbial form biofilm on the electrode surface was the foundation in bioelectrochemical systems’ normal running process. In the pioneer’s work, many genes were identified, which are crucial for the biofilm formation, such as *dgcS*, *cheY3*, *exeM*, and *bolA* gene. DgcS, a major diguanylate cyclase (DGC), catalyzed GTP to form cyclic diguanosine monophosphate (c-di-GMP), which acts as a second messenger to regulate biofilm formation (Matsumoto et al., 2021). At the same time, phosphorylated CheY3 was also observed to interact with DGCs and co-regulate the biofilm formation (Boyeldieu et al., 2020). ExeM, an extracellular nuclease, was also identified as a crucial enzyme for the normal biofilm formation, activated by the metal cofactors (Ca^2+^ and Mg^2+^/Mn^2+^) (Binnenkade et al., 2018). The scientist also found that overexpression of the *bolA* gene, a transcriptional regulator, facilitates biofilm formation by regulating many related gene express processes (Silva et al., 2020). Beyond that, low concentrations of extracellular riboflavin resulted in an upregulation transcription of the ornithine decarboxylase–encoding gene *speC*, which facilitates the biofilm formation of *S. oneidensis* (Silva et al., 2020). So, except electrode surface modification, genetic manipulation can also regulate biofilm formation in a sample and stable method.

### Construct Genetic Manipulation Platform

Genetic manipulation platform development, especially non-model organism, not only removes gene editing restrictions but also broaden the product type. Fortunately, the developed synthetic biotechnology of the CRISPR-related toolset provides new possibilities for reprogramming the gene module in electroactive microorganisms. By fusing Cas9 nickase (Cas9n (D10A)) with cytidine deaminase (rAPOBEC1), Cheng et al. successfully developed the pCBEso system in *S. oneidensis*, whose double-locus simultaneous editing efficiency reached up to 87.5%. Compared with others, this system did not require double-strand break or repair templates and was successfully used for broadening carbon source utilization spectra for *S. oneidensis* (Cheng L et al., 2020). The developed CRISPR-ddAsCpf1 system achieved 100% gene repression reported by green fluorescent protein (GFP). By repressing the gene related to extracellular electron transfer, they realized the enhancement of l-lactate metabolism–related genes expression and riboflavin production, resulting in rediverting electron flux (Li et al., 2020). Ng’s group also applied CRISPR interference (CRISPRi) targeted to the genes and redirection carbon flux of *S. oneidensis*, combined with integrating gene cluster coding the glucokinase, GroELS chaperone, and ALA synthase under dual T7 promoters, targeted product (5-aminolevulinic acid) improved by 145-fold (Yi and Ng, 2021). Yang’s group successfully explored a way to produce n-butanol (160 mg/L) through engineered *S. oneidensis* MR-1. The gene modules encoding alcohol dehydrogenase, CoA transferases, and acetyl-CoA synthetase were integrated into the plasmid and worked in the bacteria (Jeon et al., 2018). These genetic manipulation platforms constructed would accelerate the robust construction of engineered strain with superior performance electrically driven carbon neutrality.

### Accelerate Electron Transfer Rate

Synthetic biotechnology can also be employed to modify and reconstitute the related pathways and regulation strategies to enhance extracellular electron transfer (EET) efficiency. The scientist developed a population-state decision system based on quorum sensing to allocate cellular resources, which change the predominant metabolic flux from growth to EET in the latter, resulting in EET enhancement up to 4.8-fold (Li et al., 2020). Dundas et al. designed a series transcriptional logic gate to regulate and control the EET flux in *S. oneidensis*, in which the EET pathway–related parts of CymA/MtrCAB were systematically modified (Dundas et al., 2018). These works provide novel and powerful methods to control and regulate the EET, which lay the foundation for developing an intelligently and effectively synthetic biotechnology tool.

The cytochrome c network is a crucial module that bridges the electron transfer between intracellular and extracellular environments, including c-Cyts CymA, bc1 complex, TorC, and SirD (Figure 5). Sun et al. constructed engineered *S. oneidensis* whose NapB, FccA, and TsdB were depleted, and *CctA* was overproduced, resulting in higher power density in MFCs (436.5 mW/m^2^, 3.62-fold than that of wild type) (Sun et al., 2021). At the same time, soluble c-type cytochromes (c-Cyts) also enhance the extracellular electron shuttling under electron acceptor–limited conditions (Liu et al., 2020). The scientists also attempted to enhance the electron transfer by focusing on another crucial factor-cAMP, for cAMP-cyclic adenosine 3′,5′-monophosphate receptor protein (CRP) regulates the multiple EET-related pathways (Vellingiri et al., 2019). Scientists constructed a cyaC-OE mutant that expresses higher intracellular cAMP concentration, five times higher than that in the wild-type strain, resulting in a two-fold higher current than the wild-type strain (Kasai et al., 2019). Cheng et al. expressed exogenous adenylate cyclase encoding gene from the *Beggiatoa* sp. in *S. oneidensis* MR-1, enhancing EET capacities (Cheng ZH et al., 2020).

## Conclusion and Perspective

On the one hand, CO_2_ emitted from fossil fuels and the productive human activity was the main factor for extreme climate change and global warming. On the other hand, CO_2_ is also a carbon source, catalyzing more valuable products. Cyanobacteria are a natural microorganism that can spontaneously convert CO_2_ to many chemicals. At the same time, synthetic biotechnology was also employed to construct engineered bacteria by introducing the heterogeneous CO_2_ fixation–related gene for carbon neutrality. Computational analysis was also another powerful tool to identify and design pathways with a more favorable thermodynamic driving force for CO_2_ fixation (Satanowski et al., 2020; Chou et al., 2021). Beyond that, electrically driven microbial and enzyme engineering was also suitable for CO_2_ fixation with high efficiency. Thus, combined synthetic biotechnology with electrochemistry possesses the full advantage of both for carbon neutrality, which can not only broaden the product scope but also harvest high energy conversion efficiency.

Whether inartificial CO_2_ autotrophic organism or a heterotrophic model organism, synthetic biotechnology tools play a crucial role in editing genes, rediverting metabolic pathways, endowing them with new functions, optimal enforcement efficiency, and most abundant resources. Based on this, scientists carried out milestone work that converts the heterotrophic organism of *E. coli* (Figure 2) (Antonovsky et al., 2016; Gleizer et al., 2019; Bang et al., 2020) and *P. pastoris* to autotroph and growth on CO_2_ (Figure 3) (Gassler et al., 2020). These great works will pave the way for the CO_2_ fixation through microorganisms and the great dream of carbon neutrality. However, the CO_2_ fixation rate was slow and insufficient to satisfy the conversion of CO_2_ to the chemical product with high efficiency. Thus, electrochemical combined with synthetic biotechnology spawned a new revolution for CO_2_ fixation efficiency. By developing a chemoenzymatic system, CO_2_ was first fixed through electrochemical to form methanol, coupling with multi-step enzymatic reaction, CO_2_ was converted to starch at last with a rate ∼8.5-fold higher than starch synthesis in maize (Cai et al., 2021). This is a typical case in fuse electrochemical synthetic biotechnology involvement, which gives attention to both efficiency and product quality. Beyond that, the success of the cell-free model is also pinning its hopes on enzymes (such as formate dehydrogenase and carboxylase), whose design and engineering can also accelerate the rate of CO_2_ fixation. Thus, some novel analytical (Zhuang et al., 2021) methods and research tools (Zhuang et al., 2020) can also show extraordinary talents in illuminating the enzyme’s catalytic mechanism.

Besides, it is also an important and promising field that equips microorganisms with highly efficient light-harvesting inorganic semiconductors to realize light-driven carbon fixation (Brown and king, 2020). By employing cadmium sulfide nanoparticles, scientists not only enable the photosynthesis of acetic acid from carbon dioxide through *Moorella thermoacetica* (Sakimoto et al., 2016) but also enhance the CO_2_ fixation pathway in *E. coli* (Hu et al., 2021)*.* Furthermore, this technique also enhances the yields of l-malate and butyrate from glucose with theoretical yields (1.48 and 0.79 mol/mol) in *E. coli* (Hu et al., 2021). So, interdisciplinary light and/or electrically driven synthetic biotechnology will shine their light on CO_2_ neutrality with more high value–added products and higher efficiency in the future.
